# Incidence of Helminthic and Viral Coinfections in Malaria Patients in the Tertiary Care Hospital Setup

**DOI:** 10.1155/2024/8529788

**Published:** 2024-03-28

**Authors:** Murad A. Mubaraki, Mubbashir Hussain, Fozia Fozia, Ijaz Ahmad, Shahid Niaz Khan, Abdul Qadir Khan, Ziaullah Ziaullah

**Affiliations:** ^1^Clinical Laboratory Sciences Department, College of Applied Medical Sciences, King Saud University, Riyadh, Saudi Arabia; ^2^Department of Microbiology, Kohat University of Science and Technology, Kohat, Pakistan; ^3^Department of Biochemistry, KMU Institute of Dental Sciences, Kohat 26000, KP, Pakistan; ^4^Department of Chemistry, Kohat University of Sciences & Technology, Kohat, Pakistan; ^5^Department of Zoology, Kohat University of Science and Technology, Kohat, Pakistan; ^6^College of Professional Studies, Northeastern University, Boston, MA, USA

## Abstract

**Introduction:**

This study determines the incidence of common viral and helminth coinfections with malaria in the tertiary care hospital set up in southern Khyber Pakhtunkhwa, Pakistan.

**Materials and Methods:**

The multidimensional research included malaria patients admitted to different hospitals of district Kohat during January and December 2021. Stool samples and blood were assembled from the patients. Giemsa-stained microscopy-positive samples were processed by the immunochromatography technique (ICT) to identify *Plasmodium* species. Common viral infections such as viral hepatitis (A, B, and C), HIV, and dengue (DENV) were analyzed by ICT kits while SARS-CoV-2 was confirmed through real-time PCR. Furthermore, the intestinal helminths were identified using the Kato-Katz thick smear method.

**Results:**

Among 1278 patients, 548 were diagnosed with malaria, 412 (75.2%) were positive for *P. vivax* infection, 115 (21%) for *P. falciparum*, and 21 (3.8%) for mixed malaria infection (*P. vivax/P. falciparum*), with a higher incidence among males (65.2%) than females (34.8%). Coinfection with helminths was positive in 215 (39.3%) malaria patients. The most common infections were caused by the *Ascaris lumbricoides* species (42.6%) followed by *Enterobius vermicularis* (31.7%) and hookworm. A total of 24.6% of malaria-positive cases were also coinfected with different viruses with higher frequencies of confection for HAV (8.2%) and DENV (6.2%), respectively. The patients revealed higher incidence of coinfections with *P. falciparum* (57%) as compared with *P. vivax* (39.2%) and mixed infections (3.7%).

**Conclusion:**

This study demonstrated that the study population exhibited a significant incidence of coinfections with intestinal helminth and viral malaria.

## 1. Introduction

In 87 malaria-endemic countries, there are expected to be 229 million malaria infections and 409,000 deaths from the malaria disease that is an ongoing threat to global public health [1]. In Pakistan, more than 3.4 million suspected cases of malaria were recorded between January and August 2022 compared to 2.6 million cases reported during the same period in 2021. Thus, Pakistan is epidemic for malaria [2]. Approximately, 170,000 cases were confirmed in the laboratory, with 77% of those cases being caused by *Plasmodium vivax* and 23% by *Plasmodium falciparum*, which is linked to the deadliest and most severe case [2].

Several factors such as agriculture and outdoor occupations expose folks to malaria as well as other vector-borne diseases. Recent years have witnessed an unanticipated increase in dengue (DENV) and chikungunya (CHKV) virus incidence in Pakistan [3]. Similarly, other viral infections such as viral hepatitis (HBV, HCV, and HAV), HIV, and most recently, SARS-CoV-2 are also quite common in different provinces of Pakistan [4]. Several studies from different countries described the concurrent spread of malaria and arboviruses and viral hepatitis [5]. Apart from viral infections, parasitic infections, particularly those due to helminths, are quite common in developing countries. According to research studies, children living in countries with low incomes may coexist with *Plasmodium* and soil-transmitted helminths [6]. The increased incidence of malaria is partly caused by the frequent coexistence of parasitic helminths in children residing in low- and middle-income countries [7]. This overlapping endemicity of malaria parasites and intestinal helminths is considered to be responsible for a high prevalence of helminth coinfections, with interactions between helminths and malaria parasites [8]. Geographical overlapping of different pathogens including helminths and prevalent disease of infection such as human immunodeficiency virus (HIV), tuberculosis, hepatitis C, and malaria virus results in alteration of host susceptibility to many other pathogens. An increasing amount of data indicates that those with helminth infections or viral infections are more susceptible than helminth-free people to develop clinical *P. falciparum* malaria [8, 9]. Confections or malaria caused by other infections might change the course of the clinical history of diseases, complicated identification, and therapy. Furthermore, the clinical presentation of malaria is similar, which can lead in an apparent inadequate response to antimalarial drugs and the misdiagnosis of coinfections. The coinfections are thought to impede the progression of malaria.

A recent decline in the health system capacity in Pakistan due to political and socioeconomic destability has led to the malaria control program and deterioration of the epidemiological surveillance [10]. The occurrence of several coinfections in malaria patients in diverse geographical areas cannot be ignored, as this tropical country has a high endemicity of malaria as well as a high prevalence of various viral and helminth diseases. Limited data is available about the frequency of viral and helminth coinfections in malaria patients from this part of the world. The improved clinical management and outcomes for malaria can be achieved by directing the diagnostic workup and gaining an understanding of the frequency, dynamics, and causes of nonmalarial fevers in hospital settings. The study will further be helpful to study epidemiological patterns of malaria in an area endemic for malaria, helminths, and viruses.

## 2. Methodology

### 2.1. Ethical Considerations

The ethical committee of KUST Kohat University of Science and Technology, Kohat, Pakistan, provided the ethical study report, which was approved (KUST/EC/1178). All the patients who continued to be included in this study gave their informed permission. The Directorate of the Malaria Control Programme, Pakistan, granted permission to carry out the study and provided pertinent data.

### 2.2. Study Area

The present investigations were carried out between January 2021 and December 2021 in different hospitals of district Kohat, Khyber Pakhtunkhwa (KP), Pakistan. In the south region of KP, the small district of Kohat has three government hospitals and five private hospitals as part of its healthcare system. The average temperature ranges from 2 to 14°C in the winter and 35 to 44°C in the summer. Malaria and all common viral infections are commonly reported in the healthcare setups of these areas.

### 2.3. Study Population

A cross-sectional study included patients of different age groups who visited different hospitals in the study areas with suspected malaria. The inclusion criterion was any febrile patient having fever and shivering, visiting the hospitals of Kohat for fever treatment, and interested to join the study. The study only included patients who had been malaria diagnosed using microscopic analysis of thin and thick blood smears, followed by ICT/RDT for identification of the *Plasmodium* species. The detailed physical examination and standard clinical evaluation of each participant were carried out by a consultant physician in the tertiary care hospital of the study district. History related to presence of viral infections if any previously diagnosed supported by lab reports was also recorded. Sociodemographic data were obtained from the predesigned questionnaire.

### 2.4. Specimen Collection and Processing

To perform the microscopy and ICT test for viral infections and malaria, 3 ml of whole blood was taken into anticoagulated EDTA tubes. Stool samples were collected from patients by instructing them directly or to parents/guardians if the child is to place a small amount (1 teaspoon) of stool into sterile plastic containers.

#### 2.4.1. Lab Diagnosis of Malaria

*(1) Detection of Malaria Parasites by Microscopy*. Giemsa (10%) was used to prepare and stain thin and thick blood films, which were then analyzed using the aforementioned techniques [11]. The parasite density was obtained by multiplying the actual count of white blood cell of patients by the number of parasites, which was divided by 200 [12].

*(2) ICT/RDT for Detection of Plasmodium Species*. An immunochromatographic rapid diagnostic test (RDT) kit (the Malaria Pf/Pv Ag Rapid Test; Heilen®; Zhejiang Orient Gene Biotech Co, Ltd.) was purchased and used to analyze the blood samples from the malaria-positive microscopy patients.

#### 2.4.2. Stool Examination for Intestinal Helminths

The Kato-Katz thick smear method was used for preparing stool samples for microscopic inspection following their collection in labeled wide-mouth screw-capped containers [12]. In order to calculate the density of eggs, the number of eggs on each slide was multiplied by 24 to get an egg per gram of faeces (epg) [13].

#### 2.4.3. Viral Coinfections Evaluation

Commercial ICT/RDT kits were used for dengue (NS1, BASECHEK, Inc. USA), chikungunya (IgM/IgG, USA), HAV (IgG/IgM, Healstone, USA), HBV (HBsAg, Healstone, USA), HCV (IgG/IgM, Healstone, USA), and HIV (IgG/IgM, USA) following the guidelines of manufactures. The nasal swab was also obtained from patients and sent to the reference COVID-19 lab, KDA Hospital Kohat, for detection of SARS-CoV-2 by real-time PCR analysis. Viral hepatitis infection (A, B, or C) and HIV positive by ICT/RDT were further confirmed by the enzyme-linked immunosorbent assay (ELISA) using protocols recommended by the manufacturer. The HBsAg SURASE B-96 ELISA kit was used to conduct ELISA testing in accordance with the described method. The direct sandwich ELISA method was employed to qualitatively detect HBsAg in human serum or plasma. An indirect ELISA kit for HCV was used to detect HCV antibodies. HIV was identified using an ELISA (Vironostika Ag/Ab) test.

### 2.5. Statistical Analysis

The SPSS Statistics for Windows v.25 was used to process the data by applying *t*-Student, Fisher's exact, and Chi square tests. The odds ratio (OR) for problems based on coinfection was calculated with a 95% CI. A statistically significant *p* value was to be <0.005.

## 3. Results

**Figure 1:**
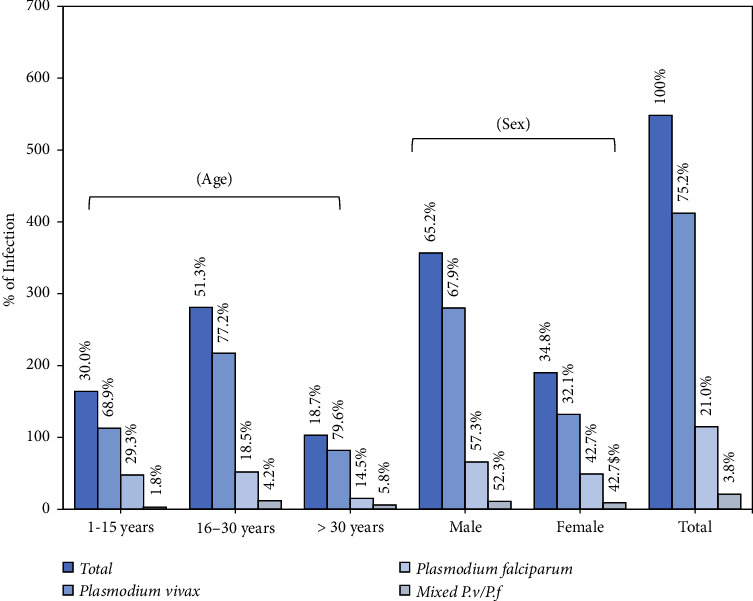
Frequency of *P. vivax*, *P. falciparum*, and mixed infection in Kohat district.

Over one-year period (January 2021–December 2021), 548 out of 1278 sampled participants tested positive for malaria that was confirmed by microscopy and ICT, resulting in a frequency of 25%. Among these 548 patients diagnosed with malaria, 412 (75.2%) were positive for *P. vivax* infection, 115 (21%) *P. falciparum*, and 21 (3.8%) mixed malaria infection (*P. vivax/P. falciparum*). The frequency was higher among males (357/547 (65.2%) than females (190/547, 34.8%). A statistically significant correlation (*p* < 0.005) was found between the gender and the prevalence of malaria.  Figure 1 shows that the age group between 1 and 15 years old had a greater incidence of malaria (164, 30%) than other age groups. There was no statistically significant correlation found between age and the incidence of malaria (*χ*^2^ = 0.5112, *p*=0.226).

### 3.1. Frequency of Intestinal Helminths Coinfections

**Figure 2:**
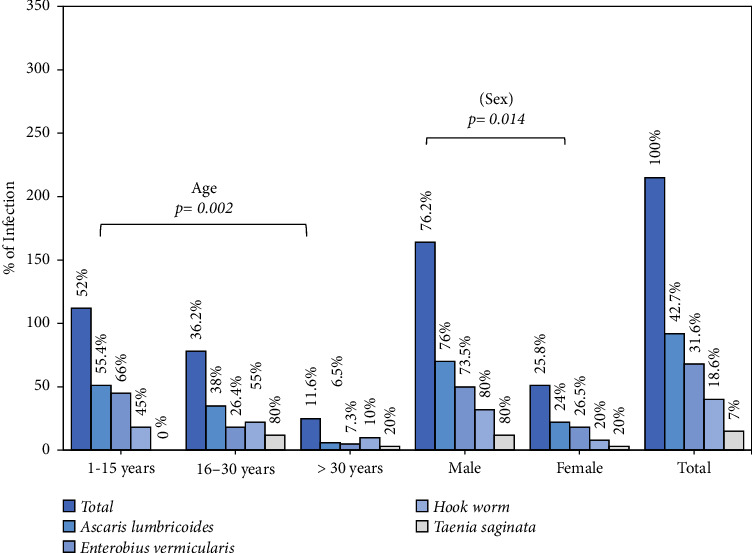
Incidence of intestinal helminths among age and sex groups.

**Figure 3:**
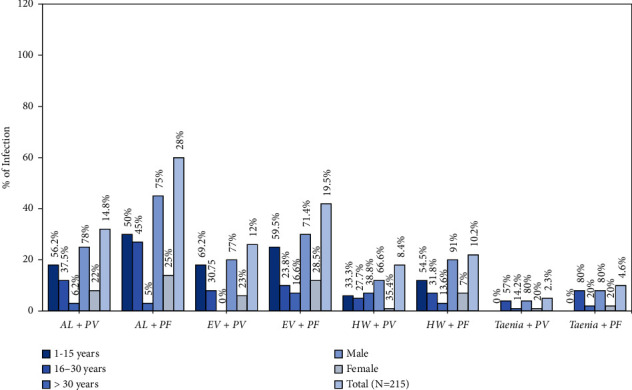
Incidence of *P. vivax* and *P*. *falciparum* malaria and helminths coinfection (PF  =  *P*. *falciparum*; PV =  *P*. *vivax*; HW =  hook worm; AL = *Ascaris lumbricoides*).

Among the 547 malaria positive participants, 215 intestinal helminths exhibit positive that demonstrate 39.3% of an incidence. The frequency of helminths was higher (*p*=0.003) in the children age group of 1–15 years (22/116 (19.0%)) compared to other age groups as shown in Figure 2. The males incidence of infection (27/197, 13.7%) was higher than that of females (22/214, 10.3%). In addition, a significant correlation was found between gender and the incidence of helminthes (*χ*^2^ = 8.142, *p*=0.005). The most prevalent infection was caused by the *Ascaris lumbricoides* species (42.7%), which was followed by *Enterobius vermicularis* (31.6%) and hookworm (18.2%). Lowest frequency was recorded for *Taenia* species (7%). A higher frequency of helminths was found in the age group 1–15 years (112, 52%; *p*=0.002). More than one species of helminths infection at a time was not part of the present study. All malaria-positive children of age group 1–15 were coinfected with helminths. The frequency of different helminths in *vivax, falciparum*, and mixed cases are shown in Figure 3 with the highest incidence of *Ascaris lumbricoides* associated with *falciparum malaria*.

### 3.2. Incidence of Viral Infections Coinfections

**Figure 4:**
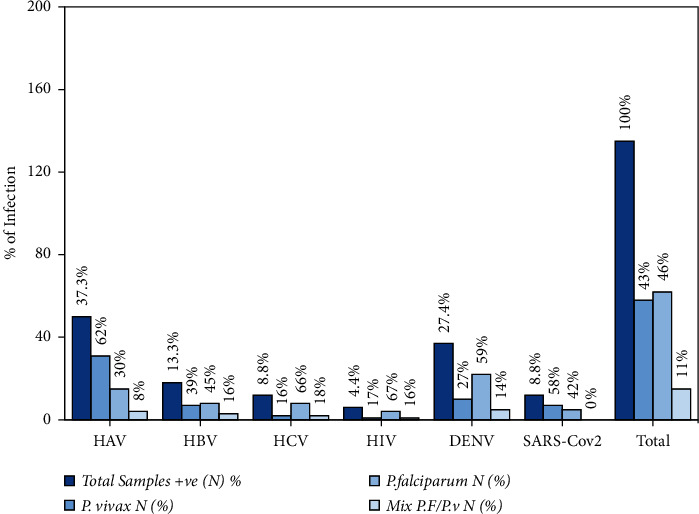
Incidence of different viral infections in *P. vivax* and *P. falciparum* malaria.

A total of 135 (135/548, 24.6%) malaria-positive patients were also coinfected with different viruses. Higher frequencies of confection were recorded for HAV (45/135, 8.2%) and DENV (34/548, 6.2%), respectively. The patients showed higher incidence of coinfections with *P. falciparum* (*n* = 77/135, 57%) in contrast with *P. vivax* (*n* = 53/135, 39.2%) and mixed infections (*n* = 5/135, 3.7%) (OR = 3.12; 95% CI, *p*=0.024). Simultaneous coinfection with was not determined as it was not part of the present study. Risk factors associated with coinfections of viruses with malaria were also studied. The lowest rate of coinfection was recorded in the age group 1–10 years in which only 1 (2.2%) patient was found coinfected with HAV. The highest rate (47/135, 34.8%) of coinfection was recorded in the age group 21–30 years. Although no significant correlation was found in malarial patients coinfected with viruses among different age groups, in the present study, 88/135 (65.2%) of malaria patients were coinfected with different types of viruses. Among these, the highest frequency of males infected with HAV (*n* = 88) and the lowest frequency of HIV was found in female patients (*n* = 1). However, the correlation between viral infections was not significant. Of a total of 75/135, 54.3% of the patients coinfected with malaria were from rural. Different factors associated with coinfections of viruses in malaria patients are tabulated in Table 1 and Figure 4.

### 3.3. Concurrent Coinfection of Virus and Helminths in Malaria Patients

The most common helmith combinations were *P. vivax*, *Ascaris lumbricoides*, and HAV (35.6%); *P. vivax* and *E. vermicularis*, and HAV (24.3%); *P. vivax*, *A. lumbricoides*, and DENV (22.4%); *P. falciparum*, *A. lumbricoides*, and DENV (5.5%); and *P. falciparum*, hookworm, and HAV (4.8%). Similarly, *P. vivax*, *P. falciparum*, *A. lumbricoides*, and DENV concurrent infection was also found (2.0%). 3/6 HIV-infected patients were also found coinfected with *P. faciparum, A. lumbricoides*, hookworm, and DENV. Children in the 15–30 age group exhibited higher rates of coinfections with helminth, viruses, and malaria (*p* < 0.001).

### 3.4. Association between Complicated Malaria and Coinfections

A confirmed complicated malaria case is one in which one or more clinical or laboratory parameters confirm hepatic, renal, pulmonary, or cerebral complication. A total of 183/215 (85.1%) patients coinfected with helminths were having complications. The most frequent complications in patients were anemia (152/183, 83%), jaundice (112/183, 61.2%), and cerebral malaria (22/183; 12%). Of the complicated cases, 81/183 (44.2%) were in the *P. vivax* infected group, 69/183 (37.7%) in the *P. falciparum*, and 8/183 (4.34%) in patients having mixed *P. vivax/P. falciparum* malaria. In 86/135 (63.7%), malaria patients with viral coinfections had complications. Among these, all the patients suffering from HIV (6/6, 100%) were having complications in terms of anemia (28/86, 32.5%), jaundice (16/86, 18.6%), and renal impairment (10/56, 11.6%).

## 4. Discussion

Reports about viral coinfections in malaria patients in Pakistan have been scarce. However, numerous studies, the majority of which from Southeast Asia and Africa, document the cooccurrence of malaria and other diseases such as helminths, bacteria, and viruses [6, 8, 14–17]. To the best of our comprehension, no data exist in the research area about the coinfection of viruses and helminths in malaria patients. The coinfections clearly demonstrate the potential to exacerbate malaria and result in treatment and control failure. The current reported was carried out to investigate the malaria coinfection with common viral and helminths reported in different hospitals in the south of Khyber Pakhtunkhwa province, Pakistan.

So, the present study suggests the overall malaria incidence in the research area that was 25%. Among these, 548 patients were diagnosed with malaria, 412 (75.2%) were positive for *P. vivax* infection, 115 (21%) for *P. falciparum*, and 21 (3.8%) for mixed malaria infection (*P. vivax/P. falciparum*). The incidence rate of *P. vivax* malaria in Pakistan is around 84% for all the malaria cases reported in Pakistan while the prevalence of *P*. *falciparum* and *P*. *vivax* infections are 23% and 77% cases, respectively [18]. Malaria is particularly prevalent in tribal districts of KP, which are one of the most remoted and impoverished places of Pakistan [19, 20]. In one of the studies from Kohat, the relative risk of malaria was 4.0 times higher in local population [21]. In the present study, samples were obtained from only local population and none of the case was imported. The differences in incidence rates might be due to environmental and seasonal variation, socioeconomic status, availability of proper diagnostic and therapeutic, or the geographical difference of the study participants.

### 4.1. Helminth Malaria Coinfection

The current investigation demonstrates that the incidence of intestinal helminths coinfection in malaria patients was 39.3%, which is higher than the incidences of 32.5% in South Western Cameroon [22] and 34.2.7% in South West Ethiopia [23]. For comparison, there is no report yet published from Pakistan about the prevalence of different helminths in malarial patients; therefore, the comparison is made with similar studies published from other geographical areas. Different diagnostic methods used to detect the parasites as well as geographic variations may be the cause of these disparities. Our study area is considered a neglected area in terms of deworming programs, which may account for the higher incidence of intestinal helminths in this area. The higher prevalence of helminths malaria coinfections could also be linked to environmental contamination, poor hand-washing practices, using sewage water to irrigate crops, and also due to various factors such as environmental, socioeconomic, and behavioral factors to increased susceptibility of helminths-infected individuals to *Plasmodium* infection. The incidence of different helminths in the study were *Ascaris lumbricoides* (42.7%), *Enterobius vermicularis* (31.6%), hookworm (18.6%), and *Taenia saginata* (7%). Other helminths were not detected in the present study. Accordingly, the present study was carried out in different regions of Pakistan and other countries; *Ascaris lumbricoides* is the most prevalent and common helminth species that causes infection in both adults and children. A prevalence of 30% of *Ascaris lumbricoides* has been reported from a study conducted in the neighboring district of Waziristan [24] and 31.7% in the district Peshawar of KP, Pakistan [25]. *Taenia saginata* prevalence rate of 21% has been reported from Swat, Pakistan [26]. The results of the previous studies as well as the current study indicate that helminth infections are quite common in this part of Pakistan which is also endemic for malaria. But there are limited data available about coinfections of malaria and different helminth infections. Intestinal parasitic infections were more common in malaria patients in children aged 1–15 years and above (*p*=0.003), which is in accordance with previous reports from around the world [8, 27, 28]. This could be attributed to differences in children's exposure levels as they grow. Among different age groups, group consisting of young subjects of 1–15 years had the highest incidence rate (112/215, 51%) of coinfection although the results are statistically nonsignificant. This figure is high as compared to 26.1% reported previously in Ethiopia and lesser than 60% reported from Tanzania [29]. In the present study, the higher rate of coinfection was seen, which could be due to the fact that children have usually a higher rate of helminth infection rates due to unhygienic lifestyles in rural areas. Most of the subjects from this age group belong to rural areas with lower education rates and poor socioeconomic status.

### 4.2. Malaria Viral Coinfections

In the present study, an overall incidence rate of 24.6% of viral coinfection in malaria-positive patients was recorded. The patient showed higher incidence of coinfections with *P. falciparum* (57%) in contrast with *P. vivax* (39.2%) and mixed infections (3.7%). Most cases of viral coinfections with malaria have been reported from India [30, 31] and Africa [32].

### 4.3. Viral Hepatitis and Malaria Coinfection

In present study, an incidence of 37% was recorded for HAV coinfection with malaria. Only 3 patients were young subjects of 1–10 years while the rest of the cases were found in other age groups. This is due to the fact that malaria-positive cases in young subjects were found less frequently in current investigations. The research in Kenya also reported a low incidence of 1.7% in children coinfected with malaria and HAV [33]. HAV is endemic in Pakistan. Due to unsanitary conditions, a 60% prevalence of HAV was recorded in five major cities in Sindh, Pakistan, especially in young children aged 2 m ≤ 10 years [34]. These differences in the incidence rate in different geographical areas could be because of differences in age, sex, and race-specific rates among different regions of Pakistan. Since transmission of HAV is a feco-oral route, the water and sanitation system in the study area is deteriorated. Our results indicate that majority of the HAV infected patients were from rural areas of study district where there is usually an inadequate supply of clean drinking water.

In the present study, the incidence of HBV (14/135) and HCV (16/135) were recorded with 7 cases each for both in 31–40 years age group. The higher prevalence of viral coinfections particularly with HBV and HCV were detected in the 21–30 years age group. In another study, a high incidence of HBV that was detected in the age group of 46–60 years for male and female with the rate of infection was 25% and 23% [35], which is in accordance with the present study. The frequency of viral coinfections with malaria was higher in males as compared to females which are due to the religious and cultural values of the study area. The older malaria patients (60 years and above) exhibited the lowest infection rate. The Eastern Mediterranean area has the greatest frequency of HCV at about 2.3% [36].

### 4.4. DENV and Malaria Coinfection

The current investigation demonstrates the incidence of malaria coinfection with DENV was the second highest (27.4%) after HAV. Although there was a nonsignificant association among different age groups, the incidence rate of DENV was 25.1% (34/135). Most of the country experiences dengue and malaria outbreaks during the postmonsoon season as a result of prolonged periods of stagnant water bodies spurred on by inadequate sewage and sanitary infrastructure. In comparison to a study from Lahore, Pakistan, which reported a prevalence rate of 33%, our study demonstrated a lower coinfection incidence of DENV with malaria [37] which was done during dengue outbreak of 2012 which accounts for higher prevalence. However, the reason for lower coinfection rate in our study was that the sampling was done throughout the year irrespective of specific season or outbreak. Our results are in contrast with studies from Cameroon (8.9%) [38], India (3.4% and 3%) [39, 40], and 13.1% in Bangladesh [40]. The lower incidence of coinfection with DENV in other parts could be due to the fact that dengue outbreaks occur in these areas occasionally. Furthermore, the prevalence of DENV-malaria coinfection varies within the geographical area that depends on the sensitivity of the diagnostic methods and also local endemicity which varies from place to place. Coinfection of dengue with malaria can be troublesome in countries with high rates of malaria, like Pakistan, that have limited diagnostic capabilities because it is a routine practice to treat febrile cases as being malaria. Malaria and dengue coinfections are often ignored and mistakenly believed to be monoinfections due to their comparable clinical manifestations.

### 4.5. HIV Malaria Coinfection

In the present study, HIV coinfection was found in only 5 patients all of whom were male. Many studies have been conducted in patient of HIV infected simultaneously with plasmodium coinfection species. In one meta-analysis study done, the average prevalence of malaria among HIV-positive children, HIV-positive pregnant women, and HIV-positive adults was 39.4%, 32.3%, and 27.3%, respectively [41]. We could not find any previous study about HIV from Kohat to compare our results.

### 4.6. SARS-CoV-2 Malaria Coinfection

In the present study, 12/135 malaria patients were found coinfected with SARS-CoV-2. The coinfection of malaria in COVID-19 patients has already been reported from Pakistan [42]. In one of the studies from Sudan [43], a much greater number of malaria diagnoses than those reported in our study 270/591 (45.7%) confirmed that COVID-19 patients were observed. The possible reason of low coinfectivity in our study could be the high vaccination rate which is around 60% of the Pakistani population vaccinated. According to the results of one meta-analysis study, the total prevalence of *Plasmodium* spp. infection (364 cases) among COVID-19 patients (1,126 cases) is 11%, with a significant degree of variability [44].

### 4.7. Viral, Helminth, and Malaria Coinfection

This investigation also showed that coinfections with viruses and helminths are frequent in the research area among people suffering from malaria. Majority of the individuals mainly between 15 and 30 years infected with *P. vivax* were simultaneously disease ridden with one or more virus and helminth. There is no previous study available to compare our results of concurrent infection of malaria, helminths, and viruses. Natural concurrent infections frequently involve several parasites, including protozoa, viruses, bacteria, and helminths, in an assortment of interactions that have been documented. In infections that are mixed, the burden of one or both pathogens might be elevated, suppressed, or elevated while the other is inhibited [45]. When considering the geographic overlap between helminth-endemic areas and common infectious diseases including malaria, HIV, TB, HCV, and DENV, it appears plausible helminths change host vulnerability to numerous pathogens [36, 46]. These coinfections will result in immunosuppression and more susceptibility to the secondary viral and bacterial infection which will further deteriorate the patient's condition. In present study, we found a higher incidence of malaria in patients infected with *A. lumbricoides* followed by *E. vermicularis* and hookworm. According to earlier research, *Ascaris lumbricoides*, hookworm infection, and malaria are closely associated [47, 48]. The presence of a positive correlation between helminth infection and malaria suggests multiple underlying mechanisms, which are supported by various shared risk factors such as behavioral, socioeconomic, and environmental factors. These factors may increase the susceptibility of individuals infected with helminths to infection with *Plasmodium* [49].

### 4.8. Complications in Malaria Coinfection

The incidence of complications in malaria patients either infected with helminths and viral infections were 85.1% and 63.7%, respectively. Anemia was the most frequent complication found in helminth infections. In one of the studies from Africa, 35.1% of children positive for malaria and coinfected with helminths were found to be anemic [50]. Cerebral malaria was found common in *P. falciparum* malaria patients either infected with helminths or viruses. Our findings are in accordance with previously reported data [51].

### 4.9. Shortcomings of the Study

The incidence of the infections might be miscalculated because the diagnostics carried out on malaria patients in the present study for finding out viral coinfections were mainly using serological tests. Only malaria positive patients were targeted by screening them with microscopy followed by *Plasmodium* species identification by RDT. All malaria positive patients were then further screened by RDT methods for presence of abovementioned viral infections after taking history and also correlating malaria with the presence of pre-existing viral infection. In Pakistan, it is classically diagnosed by microscopy and *Plasmodium* species are confirmed by RDT as per recommended by National Malaria Control Program. Although RDTs are less sensitive and specific than ELISA and molecular techniques like PCR, yet these are used in the present study because these are the first tests done for screening of viral infections in developing country like Pakistan where access to molecular techniques is limited due to economic constraints. Only SARS-CoV-2 has been confirmed locally from the Government-provided facility of RT-PCR.

## 5. Conclusions

To the extent of our understanding, the current study was the first report from Southern Khyber Pakhtunkhwa, Pakistan, targeting the simultaneous occurrence of helminths and viruses in malaria patients. Higher rates of coinfections of helminths and viral infections are recorded during the present study. These coinfections if not diagnosed on time may result in higher rates of complicated cases that are often more difficult to treat, resulting in an increased level of socioeconomic burden in areas with compromised health infrastructure. Further research is necessary since the relationships between helminth infections and malaria that were identified in this study were not definitive. A similar type of study may be conducted in hospital settings of other major cities of Pakistan using sensitive and specific techniques such as ELISA and PCR to find out the exact picture of coinfections in malaria patients. It is essential for physicians to be aware of the coinfection of malaria with pathogens for proper therapeutic management of malaria in endemic areas.

## Figures and Tables

**Table 1:** Incidence of malaria-viral coinfections in district Kohat and associated risk factors.

Variable	Coinfection with viral infections (*N* = 135)						
	Coinfection positive	HAV (*N* = 45)	HAB (*N* = 14)	HCV (*N* = 16)	HIV (*N* = 6)	SARS-CoV- 2 (*N* = 19)	DENV (*N* = 34)
*Age (years)*							
1–10	3	1 (2.2)	0 (0)	0 (0)	0 (0)	0 (0)	2 (6)
11–20	23	12 (26.6)	2 (14.2)	1 (6.2)	0 (0)	2 (10.5)	6 (17.6)
21–30	47	18 (40)	3 (21.4)	3 (18.5)	3 (50)	6 (31.5)	12 (35.2)
31–40	35	8 (18)	7 (50)	7 (43.7)	2 (33.3)	5 (26.3)	8 (23.5)
41–50	15	2 (4.4)	1 (7.1)	2 (12.4)	0 (0)	4 (21)	6 (17.6)
Above 50	11	4 (8.8)	1 (7.1)	3 (18.5)	1 (16.6)	2 (10.5)	0 (0)

*Gender*							
Male	88	32 (71)	10 (71.4)	12 (75)	5 (83.7)	13 (68.4)	19 (56)
Female	57	13 (29)	4 (28.6)	4 (25)	1 (15.3)	6 (31.6)	15 (44)

*Resident*							
Urban	37	17 (37.7)	8 (57)	9 (56.2)	6 (100)	15 (79)	20 (59)
Rural^*∗*^	75	28 (62.3)	6 (43)	7 (43.8)	0 (0)	4 (21)	14 (41)

*Education status*							
Educated	47	25 (55.5)	4 (28.5)	5 (31.2)	1 (16.6)	9 (47.3)	16 (47)
Noneducated	88	20 (44.5)	10 (71.5)	11 (68.8)	5 (83.4)	10 (52.7)	18 (53)

*Financial status*							
Poor^*∗*^	95	35 (77.7)	11 (78.5)	12 (75)	5 (100)	12 (63)	24 (70.5)
Rich	37	10 (22.3)	3 (21.5)	4 (25)	0 (0)	7 (37)	10 (29.5)

*Presence of domestic animals*							
Present	30	10 (22.3)	2 (14.2)	1 (6.2)	0 (0)	2 (10.5)	12 (35.2)
Absent	105	35 (77.7)	12 (85.8)	15 (93.8)	5 (100)	17 (89.5)	22 (64.8)

^*∗*^Statistically significant.

## Data Availability

All the data generated during the research are already included within the article.
